# Metabolomics Analysis of PK-15 Cells with Pseudorabies Virus Infection Based on UHPLC-QE-MS

**DOI:** 10.3390/v14061158

**Published:** 2022-05-27

**Authors:** Panrao Liu, Danhe Hu, Lili Yuan, Zhengmin Lian, Xiaohui Yao, Zhenbang Zhu, Xiangdong Li

**Affiliations:** 1 Jiangsu Co-Innovation Center for Prevention and Control of Important Animal Infectious Diseases and Zoonoses, College of Veterinary Medicine, Yangzhou University, Yangzhou 225009, China; liupanrao@163.com (P.L.); hdh768@163.com (D.H.); yll96213@163.com (L.Y.); lian_zm@126.com (Z.L.); xiaohuiyao1995@163.com (X.Y.); 007583@yzu.edu.cn (Z.Z.); 2 Joint International Research Laboratory of Agriculture and Agri-Product Safety, The Ministry of Education of China, Yangzhou University, Yangzhou 225009, China

**Keywords:** pseudorabies virus, metabolomic analysis, UHPLC-QE-MS, PK-15 cells

## Abstract

Viruses depend on the metabolic mechanisms of the host to support viral replication. We utilize an approach based on ultra-high-performance liquid chromatography/Q Exactive HF-X Hybrid Quadrupole-Orbitrap Mass (UHPLC-QE-MS) to analyze the metabolic changes in PK-15 cells induced by the infections of the pseudorabies virus (PRV) variant strain and Bartha K61 strain. Infections with PRV markedly changed lots of metabolites, when compared to the uninfected cell group. Additionally, most of the differentially expressed metabolites belonged to glycerophospholipid metabolism, sphingolipid metabolism, purine metabolism, and pyrimidine metabolism. Lipid metabolites account for the highest proportion (around 35%). The results suggest that those alterations may be in favor of virion formation and genome amplification to promote PRV replication. Different PRV strains showed similar results. An understanding of PRV-induced metabolic reprogramming will provide valuable information for further studies on PRV pathogenesis and the development of antiviral therapy strategies.

## 1. Introduction

The pseudorabies virus (PRV) is a member of the *Herpesviridae* family, which causes Aujeszky’s disease (AD) in pigs [1]. Pigs are the main host of PRV, and pigs of different ages can be infected with PRV. AD leads to high mortality and symptoms related to the central nervous system in piglets, respiratory disease in adult pigs, and decreased reproduction in sows, which have resulted in great economic losses for the pig industry [2]. PRV also infects other mammals, such as ruminants, carnivores, rodents, and even humans [3,4,5], posing a concern for public health. PRV virions are composed of double-stranded DNA genomes, capsids, teguments, and envelopes. The genome is approximately 150 kb and encodes over 100 proteins [1]. PRV was discovered in the 1900s and then widely distributed in the world. Although PRV has been eradicated in some Western countries (such as the United States, Germany, and Canada), it is still prevalent in many countries [6]. The Bartha K61 strain is one of the classically attenuated PRV strains, known as Bartha K61, which was isolated in 1961 and attenuated by a series of passages in embryos and chicken cells [7]. As an attenuated live vaccine, Bartha K61 can induce effective immune responses against PRV in pigs [8,9]. However, in 2011, PRV variant strains emerged in Northern China, then caused appalling outbreaks in swine farms, including in PRV-vaccinated swine farms [10,11]. Although much progress has been made in the research on the pathogenesis of PRV, the detailed mechanisms of the interaction between PRV and host cells remain unclear.

Metabolomics is a new subject developed after genomics and proteomics. Nowadays, metabolomics has been applied to disease diagnosis, pharmaceutical research and development, nutrition science, environmental science, botany, and other fields closely related to human health [12,13]. Metabolomics focuses on low-molecular-weight metabolites (MW < 1 KD, such as sugars, lipids, amino acids, and vitamins) in various metabolic pathways, and it can reflect the changes in the metabolic response of cells or tissues to external stimulation or genetic modifications, which contribute to reveal the mechanism of interaction between host cells and external factors [14,15].

Viruses are intracellular parasites and cannot proliferate independently. They must hijack and rely on the metabolic mechanisms and resources of host cells for their own replication [16,17]. Virus infection remodels the metabolic machineries in host cells to deal with the higher metabolic demands during virus replication [18,19,20]. Zika virus infection increased the glucose utilization in the tricarboxylic acid (TCA) cycle in HFF-1 cells and elevated the AMP/ATP ratios, which led to cell death [21]. Influenza virus was shown to affect host metabolic pathways to ensure the production of viral particles. Glucose uptake and aerobic glycolysis were increased, while fatty acid β-oxidation were decreased in cells infected with influenza virus [22]. Human cytomegalovirus (HCMV) increased glycolytic flux to replenish the TCA cycle, and herpes simplex virus type-1 (HSV-1) induced the elevation of pyrimidine nucleotide components [23]. In addition, SARS-CoV-2 infection induced sphingolipid metabolism reprogramming, which was required for viral replication. The levels of glycosphingolipid and sphingolipid (sphingosine, GA1, and GM3) were markedly increased in cells and the murine model after SARS-CoV-2 infection [24]. However, a decrease in cholesterol and high- and low-density lipoproteins was induced in the blood of patients with COVID-19, which may be potential markers for monitoring the disease [25]. Therefore, metabolomics is widely used as an important tool to investigate complex virus–host interactions. In this way, these studies provide an insight into the pathogenic mechanisms and novel therapeutic methods of the virus.

The metabolic alterations induced by different viruses are distinct. PRV is one of the main pathogens of pigs, thus understanding the changes of PRV in host cell metabolism is necessary. Few studies have been performed on the host metabolism of PRV. Gou et al. established that the metabolic flux derived from glycolysis, the pentose phosphate pathway, and glutamine metabolism for nucleotide biosynthesis was necessary for PRV replication [26]. The changes of PRV infection in immortalized porcine alveolar macrophages (iPAMs) on glycerolipids, fatty acyls, glycerophospholipids, and sphingolipids have also been determined [27]. In this study, we analyze the metabolic alterations in porcine kidney cells (PK-15) infected with a PRV variant strain and Bartha K61 strain using ultra-high-performance liquid chromatography/Q Exactive HF-X Hybrid Quadrupole-Orbitrap Mass (UHPLC-QE-MS). The results show that plenty of metabolites and metabolic pathways are significantly changed during PRV infection, when compared to uninfected cell groups. It suggests that those alterations may be in favor of better viral replication. These findings may be helpful to understand the host response to PRV infection and development for this disease control.

## 2. Materials and Methods

### 2.1. Cell Culture and Virus Infection

PK-15 cells were purchased from the American Type Culture Collection (ATCC) and cultured in Dulbecco’s modified Eagle medium (DMEM) (Gibco, Waltham, MA, USA) containing 10% fetal bovine serum (FBS, Thermo Fisher Scientific, Waltham, MA, USA) at 37 °C with 5% CO_2_. PRV variant strain JS21 (abbreviation of PRV-G) and PRV Bartha K61 strain (GenBank accession no. JF797217; with abbreviation of PRV-K) were preserved in our laboratory. PRV titers were determined as the median tissue culture infective doses (TCID_50_) on PK-15 cells.

### 2.2. Virus Infection

PK-15 cells were cultured overnight at 37 °C with 5% CO_2_. When the cells’ density reached approximately 80%, they were infected with PRV at a multiplicity of infection (MOI) of 1 and incubated at 37 °C for 1 h. After washing with phosphate-buffered saline (PBS), the cells were incubated in DMEM supplemented with 2% FBS. PK-15 cell samples were harvested at 0, 6, 12, and 24 h post infection (h.p.i.).

### 2.3. Western Blot and Immunofluorescence Assay

PK-15 cells were infected with PRV (PRV-G or PRV-K) at MOI of 1. Cell samples were harvested at 6, 12, and 24 h.p.i. for immunoblotting analysis, which was performed as previously described [28]. Briefly, cells were lysed with 200 μL lysis buffer (Beyotime, Shanghai, China) for 15 min on ice. Following centrifugation, the supernatant of the lysates was denatured. Then, the samples were subjected to SDS-PAGE and transferred to nitrocellulose membranes (Sigma-Aldrich, Whatman, MA, USA). Following the incubation of antibodies of anti-PRV gB protein mAb (1:1000, preserved in our laboratory) and anti-β-actin (1:1000, Cell Signaling Technology, Danvers, MA, USA), the bands were visualized using an enhanced chemiluminescence reagent kit (Share-bio, Shanghai, China) and analyzed using ImageJ software.

PK-15 cells were inoculated on coverslips in the 6-well plate and infected with PRV (PRV-G or PRV-K) at MOI of 1. Cell samples were harvested at 6, 12, and 24 h.p.i. for immunofluorescence assay (IFA), which was performed as previously described [28]. Finally, the slides were placed on the cover glass with antifade mounting medium and visualized using an LSM 880 Zeiss confocal microscope (Carl Zeiss, Jena, Germany).

### 2.4. Sample Preparation and Extraction and UHPLC-QE-MS Analysis

PK-15 cells were infected with PRV (PRV-G or PRV-K) at MOI of 1. Cell samples were harvested at 0, 6, 12, and 24 h.p.i. There were approximately 1 × 10^7^ cells per sample. The samples of 0 h were used as control, named Mock group. Other groups were named G6, G12, G24, K6, K12, and K24, respectively. Three replicates per group were set. Cells were washed with precooled PBS, and the supernatant was cleared by centrifugation for 10 min at 13,000 rpm at 4 °C. The cell pellet was frozen in liquid nitrogen for 30 s. Following freeze-drying, the samples were dissolved in sterile water and ultrasound treatment in ice water. Following centrifugation at 12,000 rpm for 15 min at 4 °C, the supernatant was extracted with 1 mL of methanol/acetonitrile/water (2:2:1, *v*/*v*/*v*) containing isotope-labeled internal standard mixture. Following ultrasound treatment, the samples were incubated at −40 °C for 1 h, and centrifuged at 12,000 rpm at 4 °C for 15 min. The supernatant was used for the UHPLC-QE-MS analysis. All samples were obtained and mixed in equal amounts as quality control (QC) samples before testing. Then, experimental samples and QC samples were tested on the machine.

A UHPLC system (Vanquish, Thermo Fisher Scientific, Waltham, MA, USA) with a UPLC BEH Amide column (2.1 mm × 100 mm, 1.7 μm) coupled to a Q Exactive HFX mass spectrometer (Orbitrap MS, Thermo Fisher Scientific, Waltham, MA, USA) was used for LC-MS/MS analysis. Liquid chromatography phase A is an aqueous phase, containing 25 mmol/L ammonium acetate and 25 mmol/L ammonia water, and phase B is acetonitrile. The QE HFX mass spectrometer was used for acquiring full scan MS/MS spectra on information-dependent acquisition (IDA) mode under software (Xcalibur, Thermo Fisher Scientific, Waltham, MA, USA) control.

### 2.5. PCA and OPLS-DA Analyses

The raw data were converted to the mzXML format using ProteoWizard software. Then, the data were processed by R package analysis for peak identification, extraction, alignment, and integration. Principal component analysis (PCA) and orthogonal projection to latent structures discriminant analysis (OPLS-DA) were conducted [29,30]. The data were logarithmically (LOG) transformed and centered (CTR) formatted using SIMCA software (V16.0.2, Sartorius Stedim Data Analytics AB, Umea, Sweden), followed by PCA modeling analysis. OPLS-DA modeling analysis was performed on the first principal component and a 7-fold cross-validation in the SIMCA software was performed throughout the analysis. The R2X or R2Y (interpretability of the model for the categorical variable) and Q2 (the predictability of the model) were used to evaluate the model validity.

### 2.6. Total RNA Extraction and Quantitative Real-Time PCR (qPCR) Analysis

Cell samples were harvested at 24 h.p.i. after PRV (PRV-G or PRV-K) infection with different MOI; meanwhile, uninfected cells were used as control. Total RNAs from cell samples were extracted using TRNzol (TIANGEN, Beijing, China) and reverse transcribed to cDNA using a HiScript III 1st Strand cDNA Synthesis Kit (Vazyme, Nanjing, China), according to the manufacturer’s instructions. The primer sequences of the target genes to be detected were designed, and are shown in Table S1. ACTB was used as an internal reference gene. qPCR was performed using Universal SYBR qPCR Master Mix (Vazyme, Nanjing, China) and an ABI QuantStudio 3 Real-Time PCR (96-Well) Detection System. The reaction parameters were: 95 °C, 30 s; 95 °C, 10 s, 60 °C, 30 s, 40 cycles.; 95 °C, 15 s, 60 °C, 60 s, 95 °C, 15 s. All experiments were performed in triplicate. The mRNA levels of genes were quantified relative to ACTB using the comparative threshold cycle (2^-ΔΔCT^) method [31].

### 2.7. Statistical Analysis

The first principal component of variable importance in the projection (VIP > 1) and Student’s *t*-test (*p* < 0.05) was set as the standard to screen the differential metabolites. Then, the data was subjected to the KEGG Metabolome Database for identification of metabolites. Additionally, the metabolites were analyzed further via online statistical analysis (MetaboAnalyst, http://www.metaboanalyst.ca/ (accessed on 16 November 2021)) for identifying the altered metabolic pathways caused by PRV infection [32].

GraphPad Prism 7.0 software was used for the statistical analyses. *p*-values less than 0.05 were considered statistically significant. The values were expressed as the mean ± standard error of the mean. The significance in figures was indicated as follows: *, *p* < 0.05; **, *p* < 0.01.

## 3. Results

### 3.1. Replication of PRV in PK-15 Cells

**Figure 1:**
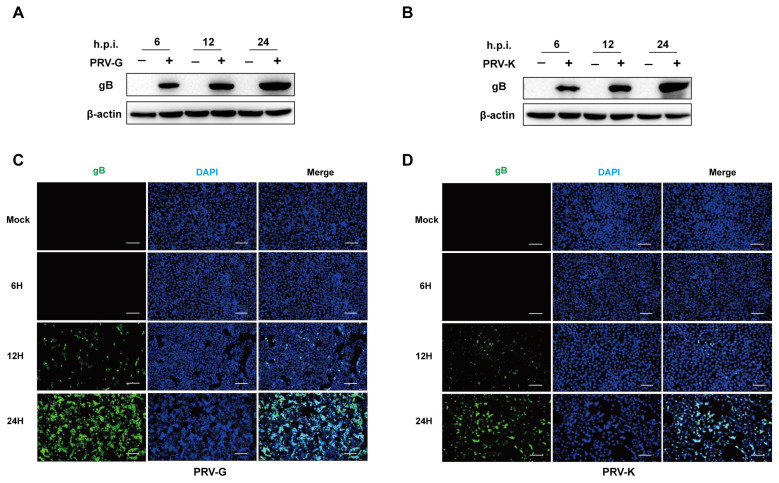
Infections of different PRV strains in PK-15 cells. (**A**,**B**) The cells were infected with PRV variant strain (abbreviation of PRV-G) and PRV Bartha K61 strain (abbreviation of PRV-K) at MOI = 1, and cell samples were collected at 6, 12, and 24 h for immunoblotting detection. (**C**,**D**) PK-15 cells were infected with PRV-G and PRV-K at MOI = 1 for 6, 12, and 24 h at 37 °C with 5% CO_2_. The expression levels of PRV-gB protein detected by IFA. Scale bars = 200 μm.

To confirm PRV replication in PK-15 cells, the cells were infected with PRV-G or PRV-K strain at MOI = 1, respectively. Additionally, the expression levels of PRV-gB protein were determined by Western blot and IFA. In the uninfected cells, there was no signal of viral protein to be detected. As shown in Figure 1A,B, the gray values of PRV-gB protein notably increase over time. Additionally, the amount and the intensity of fluorescence in PRV-infected cells were progressively strong and reached a high level at 24 h.p.i. (Figure 1C,D). These results indicate that both the PRV-G and PRV-K strains could effectively replicate in PK-15 cells within a 24 h infection.

### 3.2. Multivariate Analysis of PK-15 Cell Metabolites

**Figure 2:**
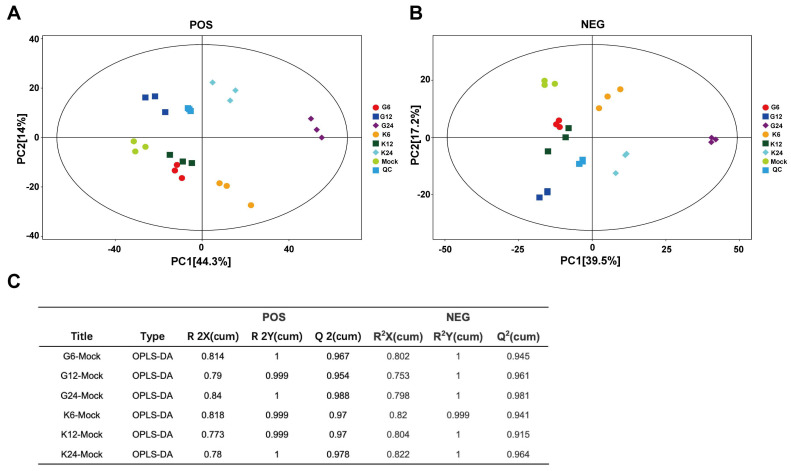
Score scatter plots of PCA and OPLS-DA of PRV-infected and uninfected cells. (**A**,**B**) Score scatter plot of the PCA model for the different infection groups versus mock group. Electrospray ionization served as the source of UHPLC-QE-MS, including positive and negative ion modes (POS and NEG). (**A**) was derived from POS and (**B**) from NEG. The lines denote 95% confidence interval Hotelling’s ellipses. (**C**) OPLS-DA model for the different PRV-strain infection group versus mock group.

The UHPLC-QE-MS used positive and negative ion (POS and NEG) switching modes and full-scan assay to screen and identify the numerous metabolites. After obtaining the data, we performed a series of multivariate pattern recognition analyses to evaluate the differences between the samples. PCA and OPLS-DA were performed to obtain more reliable information on the correlation between group differences of metabolites and experimental groups. The PCA-score scatter plot of all samples (including QC samples) is shown in Figure 2A,B. Each scatter represented a sample, and the color and shape of the scatter signed different groups. The results of the PCA score scatter plot show that all samples are in the 95% confidence interval. The OPLS-DA model for different groups versus the mock group was analyzed, and the R2X, R2Y, and Q2 of samples (POS, NEG) are shown in Figure 2C, in which the values of three parameters are close to 1. These results indicate that these different groups are clearly distinguished, and these models are efficient and reliable.

### 3.3. Differentially Expressed Metabolites during PRV Infection

**Figure 3:**
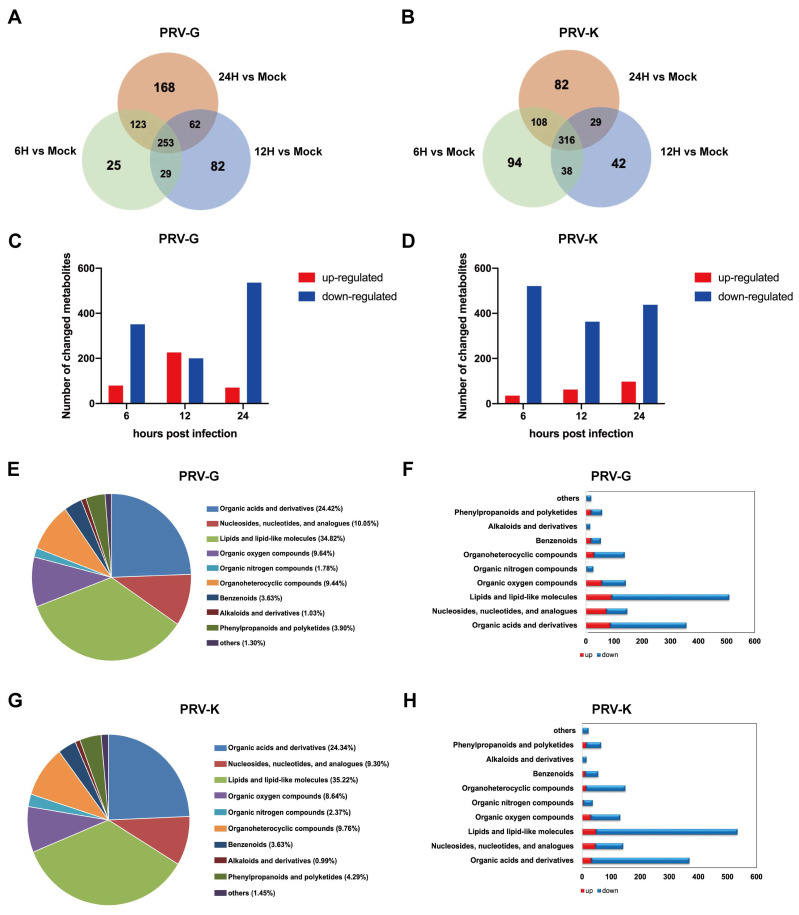
Analysis of differentially expressed metabolites in PK-15 cells infected with different PRV strains. (**A**,**B**) Venn diagrams between PRV-infected groups (6H, 12H, 24H) and mock group. (**C**,**D**) Numbers of differentially expressed metabolites upregulated (red) and downregulated (blue) in infected groups. (**E**–**H**) Pie charts and the histogram graphs showing proportions of different categories among differentially expressed metabolites in PRV-infected PK-15 cells.

Based on OPLS-DA analysis, the VIP > 1 and *p* < 0.05 were set as the standards to screen the differential metabolites. We found that a great number of metabolites were altered during PRV infection, which were summarized and shown in Venn diagrams. The numbers of differential metabolites in PK-15 cells infected with PRV-G were 430, 426, and 606 at 6, 12, and 24 h.p.i., respectively. Additionally, the numbers of differential metabolites changed by PRV-K infection were 556, 425, and 535 at different time points (Figure 3A,B). Compared to the mock group, a total of 375 and 194 metabolites were significantly upregulated in PRV-G- and PRV-K-infected cells, respectively (Figure 3C,D). Furthermore, the different changes in metabolites were due to the different times of PRV infection. In addition, these differential metabolites were classified and analyzed. As shown in Figure 3E–H, lipids and lipid-like molecules, organic acids and derivatives, nucleosides, nucleotides, analogues, and organic oxygen compounds account for nearly 80% in the PRV-infected cells. It is worth noting that, among these differential metabolites caused by both PRV strains, lipid metabolites accounted for the highest proportion: around 35%. These results suggest that the lipid metabolism of the host cell may play an important role in PRV replication.

**Figure 4:**
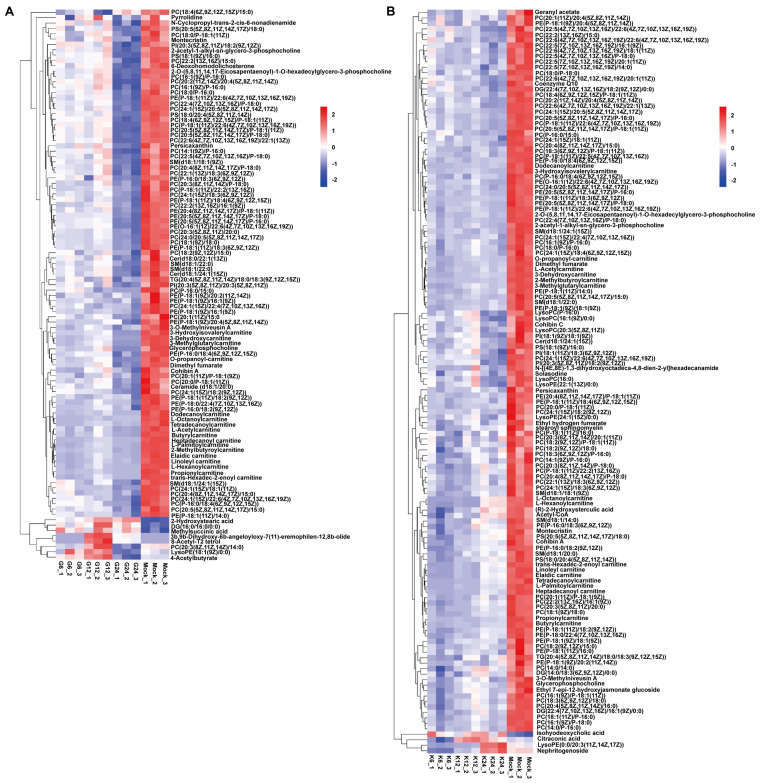
Heatmap analysis of 103 and 136 metabolites among PRV-G, PRV-K, and mock groups. Rows: metabolites; columns: samples. The color of each rectangle represents the relative level of the differential metabolites. Red: upregulated; blue: downregulated. (**A**) PRV-G; (**B**) PRV-K.

To study the crucial metabolites related to the PRV replication process, common differential metabolites among the three comparisons, i.e., 6 h.p.i. vs. mock, 12 h.p.i. vs. mock, and 24 h.p.i. vs. mock were screened. A total of 103 and 136 metabolites were obtained in PRV-G and PRV-K groups, respectively, and presented in the heatmap of hierarchical clustering analysis (Figure 4). As expected, the metabolites changed along with the virus infection. Many metabolites were significantly downregulated after PRV infection, especially lipids and lipid-like molecules. Glycerophospholipids and sphingolipids are important phospholipid molecules. Glycerophospholipids are divided into many categories according to the substitution groups, such as phosphatidylglycerol (PG), phosphatidylcholine (PC), phosphatidylserine (PS), phosphatidylethanolamine (PE), phosphatidylinositol (PI), and cardiolipin (CL). During the early stage of PRV infection, some of the glycerophospholipids increased. At 24 h.p.i., PRV-G infection induced a decrease in the levels of 78 species of glycerophospholipids, including 53 species of PC, 18 species of PE, 3 species of PS, and 2 species of PI and others (Figure 4A and Table S2). Meanwhile, PRV-K infection caused a decrease in the levels of 95 species of glycerophospholipids, including 64 species of PC, 21 species of PE, 3 species of PS, and 3 species of PI and others (Figure 4B and Table S3). In addition, few species of sphingolipids significantly decreased in PRV-G- and PRV-K-infected cells, respectively. These results indicate that, in the late stage of virus infection, PRV needs to consume a large amount of lipids in the host cell to ensure its replication.

### 3.4. Metabolic Pathway Analysis of Metabolites

**Figure 5:**
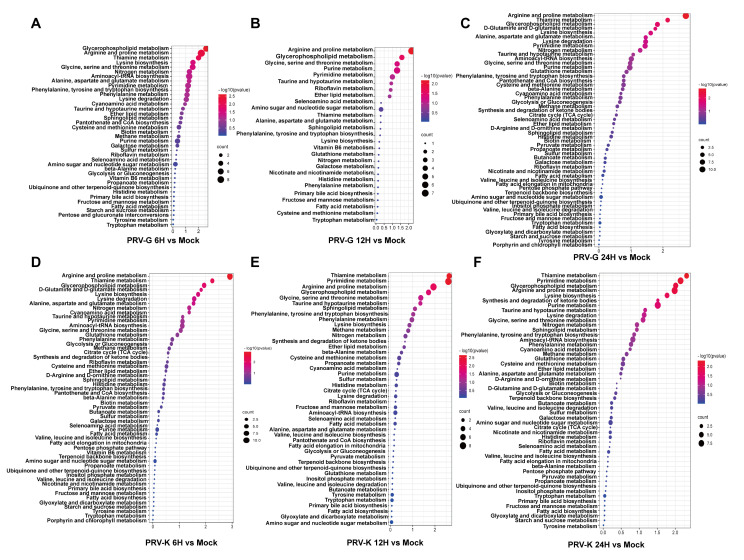
The KEGG-enrichment pathway analysis of differentially expressed metabolites for PK-15 cells infected with different PRV strains in different time courses. (**A**–**C**) PRV-G (6H, 12H, 24H); (**D**–**F**) PRV-K (6H, 12H, 24H).

These differential metabolites were annotated by using the KEGG Metabolome Database and further comprehensive analysis, including enrichment analysis and topological analysis, was conducted to find the metabolic pathways with high correlations. The results are shown in a bubble plot (Figure 5). In PRV-G vs. mock, differential metabolites were mainly enriched in arginine and proline metabolism; glycerophospholipid metabolism; glycine, serine, and threonine metabolism; purine metabolism; pyrimidine metabolism; and sphingolipid metabolism (Figure 5A–C). For PRV-K vs. mock, the metabolic pathways of the differential metabolites contained thiamine metabolism, purine metabolism, arginine and proline metabolism, glycerophospholipid metabolism, pyrimidine metabolism, and sphingolipid metabolism (Figure 5D–F).

**Figure 6:**
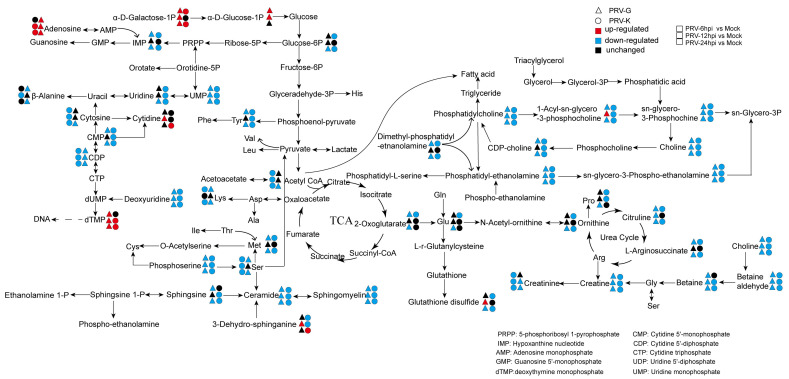
Schematic overview of altered metabolic pathways in PK-15 cells infected with different PRV strains. The metabolites were shown in different colors according to their changes. Black: unchanged; red: upregulated; blue: downregulated.

In summary, the results present more visualized profiles of the metabolite changes in PK-15 cells infected with two different PRV strains (Figure 6). The metabolic pathway contained a TCA cycle, lipid metabolism, amino acid metabolism, purine, and pyrimidine metabolism. The levels of adenosine at 12 and 24 h.p.i. in PRV-G/PRV-K-infected cells were more upregulated than that in the mock group as well as the levels of dTMP, which indicated that they may be required in the virus replication cycle. Adenosine is an important intermediate for the synthesis of adenosine triphosphate (ATP), adenine, and adenylate. Additionally, dTMP is a basic unit for deoxyribonucleic acid, which is the material basis for DNA synthesis. The major metabolic pathways of glycerophospholipids and sphingolipids and fatty acids during PRV infection were also reprogrammed, and the levels of PC, PE, ceramide, and sphingomyelin were consumed with the PRV replication process. Additionally, some amino acids were altered after PRV infection. These results indicate that two different PRV-strain infections led to the metabolic reprogramming of PK-15 cells to benefit self-replication.

### 3.5. Validation of Metabolomic Data by qPCR

**Figure 7:**
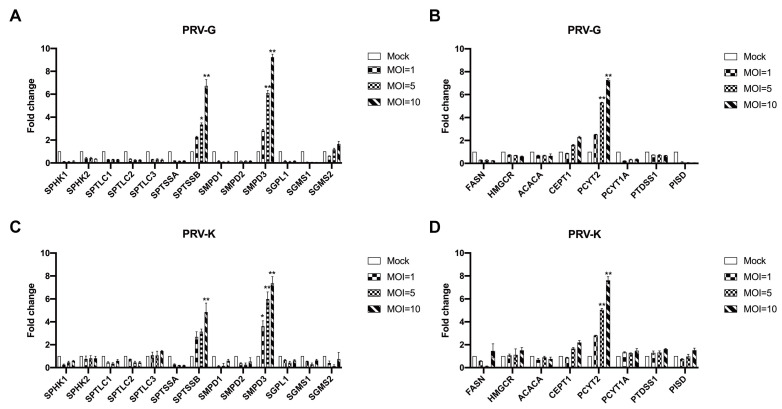
The mRNA levels of sphingolipid- and glycerophospholipid-metabolism-related enzymes after different PRV-strain infections. PK-15 cells were harvested with different MOI (1, 5, 10) at 24 h.p.i after PRV (PRV-G or PRV-K) infection, while non-infected cells were used as control. The mRNA levels of different enzymes were determined by qRT-PCR. β-actin was used as an internal reference gene. (**A**,**B**) PRV-G; (**C**,**D**) PRV-K. The significance in the figure was indicated as follows: *, *p* < 0.05; **, *p* < 0.01.

Given the possible roles of lipid metabolism during PRV infection, PK-15 samples were harvested at 24 h after PRV (PRV-G/PRV-K) infection with different MOI (1, 5, and 10, respectively) to further validate the metabolomic data. The mRNA levels of enzymes related to glycerophospholipid metabolism and sphingolipid metabolism were analyzed by qRT-PCR (Figure 7), including sphingosine kinase (SPHK1/2), sphingomyelin synthase (SGMS1/2), serine palmitoyltransferase small subunit A/B (SPTSSA/B), sphingomyelin phosphodiesterase 1,2,3 (SMPD1/2/3), fatty acid synthase (FASN), 3-hydroxy-3-methylglutaryl-CoA reductase (HMGCR), phosphate cytidylyltransferase (PCYT1A/2), and phosphatidylserine decarboxylase (PISD). We found that SPTSSB, SMPD3, and PCYT2 were significantly increased in PRV-G-infected PK-15 cells (Figure 7A,B). Moreover, the PRV-K strain could apparently upregulate these three genes (Figure 7C,D). These results suggest that some pathways of lipid metabolism in host cells are promoted during PRV infection to facilitate viral replication.

## 4. Discussion

Recently, a growing number of studies on the combination of metabolite profiling with disease have been reported [33,34]. Through the detection and analysis of endogenous small molecules in cells, researchers evaluated the biochemical differences between healthy and pathological organisms to obtain an insight into the pathology, etiology, and possible treatment options of diseases. In our study, we analyzed the metabolomic profiles of PRV-infected PK-15 cells based on UHPLC-QE-MS, and these findings provide new viewpoints on the interactions between PRV and host cells, which will help future studies on PRV.

This study involved a global metabonomic analysis of PK-15 cells that were infected with two different strains of PRV. Both OPLS-DA and the heatmap of hierarchical clustering analysis indicated that, compared to the mock group, PRV-infected cells showed significantly different metabolic profiles. We determined that PRV infection broke the metabolic homeostasis of PK-15 cells, caused metabolic reprogramming, and significantly affected the metabolism of lipid metabolism and nucleotides metabolism (Figure 6). The alterations of these metabolites and pathways reflected the cellular responses to PRV infection or the nutritional needs in virus replication.

The metabolic alterations caused by the infection of two different strains of PRV were different, which were caused by the characteristics of the virus itself. The sequence homology of the two strains was 96%, but the pathogenicity was significantly different. Following two different strains of PRV infection, PRV-G could cause a more serious typical cytopathic effect than that caused by PRV-K 24 h post infection. Therefore, PRV-infected cells showed strain-specific metabolic characteristics. However, there were some overlaps between these pairwise comparisons in the metabolic pathways (Figure 5), such as glycerophospholipid metabolism, sphingolipid metabolism, purine metabolism, and pyrimidine metabolism. Lipids are the structural basis of the cell biofilm, and are biologically active molecules, which participate in a variety of cellular processes and immune functions. We have known that palmitoyl-oleoyl-phosphatidylglycerol and PI inhibited inflammatory sequelae and the infection of respiratory syncytial virus and influenza A virus, by destroying the binding of virus particles to plasma membrane receptors of host cells [35]. Flaviviruses also increased lipid synthesis to expand the surface area of membranes for better replication [36]. Dengue virus (DENV) infection required the manipulation of cellular fatty acid synthesis and cholesterol biosynthesis and transport [37,38]. It has been previously found that sphingolipids were essential for the successful completion of the viral life cycle, which is involved in attachment, membrane fusion, intracellular replication, assembly, and release of several viruses [39,40,41]. In our study, as an enveloped virus, PRV also required a large amount of lipids to participate in the formation and release of virions. Consistently, we found that many lipids (including glycerophospholipids and sphingolipids) were consumed in the late stage of virus infection (Figure 4 and Figure 6). Purine metabolism and pyrimidine metabolism are the basic steps for nucleotide synthesis. In COVID-19 patients, some metabolites related to purine metabolism show an upward or downward trend compared to healthy controls, and correlation analysis showed a close correlation between these metabolites and proinflammatory cytokines/chemokines [42]. Previous studies have been reported to inhibit DENV replication with inhibitors (methotrexate and floxuridine) of the thymidine synthesis pathway [43]. Tiwari SK et al. utilized nucleoside metabolic inhibitors fluorouracil and floxuridine to inhibit Zika virus in human microglial cells [44]. Following PRV infection, nucleotide metabolism was markedly changed, suggesting an unknown role in PRV replication (Figure 6). This may expand the novel possibilities for the development of antiviral therapies.

Few studies have been performed on the host metabolism of PRV. Gou et al. explored the metabolic networks in PK-15 cells infected with PRV using gas chromatography-mass spectrometry (GC-MS) analysis. They reported that the metabolic flux derived from glycolysis, the pentose phosphate pathway, and glutamine metabolism for nucleotide biosynthesis was necessary for PRV replication [26]. In addition, Yao et al. indicated changes of PRV infection in iPAM on glycerolipids, fatty acyls, glycerophospholipids, and sphingolipids [27]. Our results are somewhat different from the previous two reports due to differences in the testing methods (GC-MS vs. LC-MS) and cells (iPAM vs. PK-15).

In our results, we found the mRNA level of PCYT2 was significantly increased in PK-15 cells infected with two PRV strains (Figure 7). PCYT2 is an important enzyme for the biosynthesis of PE from ethanolamine and diacylglycerol. Previous studies have shown treatment of PRV-infected PK-15 cells with meclizine, an inhibitor of PCYT2, led to decreased PRV infection and replication [28]. The result showed that glycerophospholipid metabolism was essential for PRV replication, but the mechanism was unclear.

Other herpesviruses, such as HSV-1, HCMV, Kaposi’s sarcoma-associated herpesvirus (KSHV), and Epstein–Barr virus (EBV), have shown a notable ability to reprogram the host’s metabolism for viral replication. HSV-1 infection led to increased levels of phosphoenolpyruvate, deoxypyrimidines, and pentose phosphate pathway intermediates [23]. Treatment with inhibitor of glucose metabolism or nucleoside analogs decreased the cell-to-cell spread and production of HSV [45,46]. HCMV infection markedly increased glucose uptake and glycolysis flux and promoted flux through the TCA cycle and fatty acid biosynthesis pathway [23,47]. Following the inhibition of fatty acid biosynthesis by drugs, the level of HCMV replication was suppressed [47]. KSHV caused changes in many metabolites of glycolysis, the pentose phosphate pathway, amino acid metabolism, and lipogenesis [48]. Moreover, latent KSHV-infected endothelial cells depended on glutamine and glutaminolysis for survival [49]. This evidence showed that metabolic changes caused by virus infection played an important role in viral replication.

In conclusion, the metabolic profiles of PK-15 cells infected with different PRV strains were analyzed to display the metabolic changes by UHPLC-QE-MS. There were significant differences in lipid metabolism and nucleotide metabolism between the PRV-infected groups and the mock group. The ability of viruses to actively modulate host metabolism is crucial for the successful completion of the viral life cycle. Many inhibitors of lipid metabolism and nucleotide metabolism are already used against some viral infections; therefore, identifying metabolic targets for antiviral therapy may be a promising strategy. Our study provides much information for a further understanding of PRV pathogenesis and drug intervention for disease control.

## Data Availability

The metabolomic data is available with the link: https://pan.baidu.com/s/1SRndDrNqckJ7DxofO5zWeA (Password: hv45, accessed on 24 May 2022).

## Supplementary Materials

The following supporting information can be downloaded at: https://www.mdpi.com/article/10.3390/v14061158/s1: Table S1. Primer sequences used for qPCR; Table S2. Identification and characterization of the metabolites in PK-15 cells infected with PRV-G; Table S3. Identification and characterization of the metabolites in PK-15 cells infected with PRV-K.
