# Supplementary material for: Metabolomics Analysis of PK-15 Cells with Pseudorabies Virus Infection Based on UHPLC-QE-MS
Source: Viruses. 2022 May 27;14(6):1158. doi: 10.3390/v14061158 (PMC9229976; doi:10.3390/v14061158)
Supplement: Supplementary file 1 [file viruses-14-01158-s001.zip › Table S1.pdf]

Table S1 Primer sequences used for qPCR

Gene Name	Full name		Primer Sequence (5'-3')
SPHK1	sphingosine kinase 1	Forward	TCCAGGCCGACATTTCCTTC
		Reverse	TCAGCCCGTCTCCAGACATA
SPHK2	sphingosine kinase 2	Forward	CCCGATTGCTCCTACTGGTC
		Reverse	TCTCCCGAGACTGTGACGAT
SPTLC1	serine palmitoyltransferase long chain base subunit 1	Forward	AACCTCTTGTCCCTCCTGTCT
		Reverse	CACAAGTCCCCACACCATACT
SPTLC2	serine palmitoyltransferase long chain base subunit 2	Forward	TGCGGACACATTCTCACAGT
		Reverse	ATCCTACCACAACGACACCG
SPTLC3	serine palmitoyltransferase long chain base subunit 3	Forward	ACCACGCATCTCTTGTGCTT
		Reverse	CTGGGGCAGATACACGATGG
SPTSSA	serine palmitoyltransferase small subunit A	Forward	GGAAGCAAATGTCCTGGTTCT
		Reverse	GAATTGAACACAGTCCGCTCC
SPTSSB	serine palmitoyltransferase small subunit B	Forward	TGCTGTGCTGTCTTAGAGCC
		Reverse	CAGGCGAATGTGGATTGGGA
SMPD1	sphingomyelin phosphodiesterase 1	Forward	GGGTTCTATGCCCTTTCCCC
		Reverse	CCTCGTCGTAGAACACCTCG
SMPD2	sphingomyelin phosphodiesterase 2	Forward	TGGATTGGTGCTCAATGCCT
		Reverse	CAGGCACCATCGTACAACCT
SMPD3	sphingomyelin phosphodiesterase 3	Forward	GATGTCGGGGTCTATGGCTG
		Reverse	GAAATCAGCCAGCCAGTCCT
SGPL1	sphingosine-1-phosphate lyase 1	Forward	ATCGGGAACTGGCCTTTGAG
		Reverse	AAGCCTCCCAGACAAGCATC
SGMS1	sphingomyelin synthase 1	Forward	TGGCACGCTGTACCTGTATC
		Reverse	GGGCTTCCCAGTCTCCAAAA
SGMS2	sphingomyelin synthase 2	Forward	TTGCCTTCGTGTACGCTCTT
		Reverse	AGTTAGTGTCAGCACGACGG
FASN	fatty acid synthase	Forward	CTGCTGGACTCGCTCTTTGA
		Reverse	CTTTGCCTATGTGCTTGCCC
HMGCR	3-hydroxy-3-methylglutaryl-CoA reductase	Forward	AAAGGAGGCATTCGACAGCA
		Reverse	ATCACCTGACCTGGACTGGA
ACACA	acetyl-CoA carboxylase alpha	Forward	CCAGCACTCCCGCTTCATAA
		Reverse	GTAAGGCCAAGCCATCCTGT
CEPT1	choline/ethanolamine phosphotransferase 1	Forward	ATTTGCTGGCAGTGATTGGAG
		Reverse	GACGCTTGTTCCTGCTATGGT
PCYT2	phosphate cytidylyltransferase 2	Forward	GAGGTCAACCGCTACAAGGG
		Reverse	ACGATCTCCGTCTTCCCGT

PCYT1A	phosphate cytidylyltransferase 1A	Forward	GCCAGACGGAACCTACAGAG
		Reverse	ACAGAACTTCAGTGCTCCTTC
PTDSS1	phosphatidylserine synthase 1	Forward	ATGCTTACCTCACCGACACG
		Reverse	AGTGCTCTGCATACCACACC
PISD	phosphatidylserine decarboxylase	Forward	ATGTCCAAACCCGCCCTGAA
		Reverse	GGCCTCCTTCATGTTCACCC
ACTB	actin beta	Forward	CTCCATCATGAAGTGCGACGT
		Reverse	GTGATCTCCTTCTGCATCCTGTC
